# Supplementary material for: Diagnostic accuracy of CT angiography and CT perfusion imaging for detecting distal medium vessel occlusions: Protocol for a systematic review and meta-analysis
Source: PLoS One. 2023 Apr 10;18(4):e0284116. doi: 10.1371/journal.pone.0284116 (PMC10085008; doi:10.1371/journal.pone.0284116)
Supplement: Attachment — Submitted filename: RESPONSE TO REVIEWERS.docx [file pone.0284116.s002.docx]

**RESPONSE TO REVIEWERS**

We are grateful for the thorough and valuable manuscript review that led to a considerable improvement of the article. Below we detail the answers to the provided remarks:

Comment: **“-Please include the ongoing ESCAPE-MeVO trial (in addition to DISTAL) in the introduction”**

Answer: We have included the ESCAPE-MeVO trial in the Introduction section: “ESCAPE-MeVO trial (NCT05151172) and DISTAL trial (NCT05029414) are underway in recruiting patients to evaluate if EVT is superior to the best medical management in DMVOs.”

Comment: **“In the Methods, the studies included go to August 31st, 2022, but in Table 1, July 2022 is written. Please correct this discrepancy”**

Answer: We have corrected and updated our target months in the manuscript. Included articles will be from inception to the 31st of December 2022, selection phase of the studies will occur during the current month of March. We expect the manuscript to be ready for submission at the beginning of May 2023.

Comment: **”Please include a statement regarding data availability upon completion of the study”**

Answer: The following sentence has been included in Material and Methods section under the Ethics and dissemination subtopic: “Aggregate data from this study will be available upon reasonable request from the corresponding author”

Comment **“-Please capitalize all months, re-read the manuscript for minor typographical errors (e.g., Page 6, under Data synthesis and sensitivity analysis, "we will collect the data mentioned above items.....". The word "items" should be removed)”**

Answer: We have capitalized all months throughout the manuscript. Additionally, we have thoroughly re-read the manuscript and corrected minor typographical errors, such as removing the word "items" from the sentence you mentioned on Page 6 under Data synthesis and sensitivity analysis.

Comment **“I think it would be interesting to perform an analysis of mCTA vs. CTP, as this has been shown to be at least equivalent, if not better, at DMVO detection in several single-center studies. Since mCTA can be generated with a minimal increase in time, no increase in radiation dose, etc., it seems like a more interesting comparison than to spCTA or CTA in general.”**

Answer: We absolutely agree with the reviewer. In fact, we were planning to do that subgroup analysis although it was not written in the protocol. We included the subtopic “Subgroup analysis” in the Material and Methods section where we added the following paragraph: “We will include a subgroup analysis according to the technique used in CTA (single phase or multiphase) as different accuracy values have been suggested between both algorithms[15,16]”

15	McDonough R V, Qiu W, Ospel JM, *et al.* Multiphase CTA-derived tissue maps aid in detection of medium vessel occlusions. *Neuroradiology* 2022;64:887–96. doi:10.1007/s00234-021-02830-8

16	Ospel JM, Bala F, McDonough R V, *et al.* Interrater Agreement and Detection Accuracy for Medium-Vessel Occlusions Using Single-Phase and Multiphase CT Angiography. *AJNR Am J Neuroradiol* 2022;**43**:93–7. doi:10.3174/ajnr.A7361
